# A global, regional, and national survey on burden and Quality of Care Index (QCI) of hematologic malignancies; global burden of disease systematic analysis 1990–2017

**DOI:** 10.1186/s40164-021-00198-2

**Published:** 2021-02-08

**Authors:** Mohammad Keykhaei, Masood Masinaei, Esmaeil Mohammadi, Sina Azadnajafabad, Negar Rezaei, Sahar Saeedi Moghaddam, Nazila Rezaei, Maryam Nasserinejad, Mohsen Abbasi-Kangevari, Mohammad-Reza Malekpour, Seyyed-Hadi Ghamari, Rosa Haghshenas, Kamyar Koliji, Farzad Kompani, Farshad Farzadfar

**Affiliations:** 1 Non-Communicable Diseases Research Center, Endocrinology and Metabolism Population Sciences Institute, Tehran University of Medical Sciences, Tehran, Iran; 2 Department of Epidemiology and Biostatistics, Tehran University of Medical Sciences, Tehran, Iran; 3 Department of Biostatistics, Faculty of Paramedical Sciences, Shahid Beheshti University of Medical Science, Tehran, Iran; 4 Social Determinants of Health Research Center, Shahid Beheshti University of Medical Sciences, Tehran, Iran; 5 Student Research Committee, Kurdistan University of Medical Sciences, Sanandaj, Iran; 6 Division of Hematology and Oncology, Children’s Medical Center, Pediatrics Center of Excellence, Tehran University of Medical Sciences, Tehran, Iran; 7 Endocrinology and Metabolism Research Center, Endocrinology and Metabolism Clinical Sciences Institute, Tehran University of Medical Sciences, Tehran, Iran

**Keywords:** Hematologic malignancies, Hodgkin lymphoma, Leukemia, Multiple myeloma, Non-hodgkin lymphoma, Quality of Care Index

## Abstract

**Background:**

Hematologic malignancies (HMs) are a heterogeneous group of cancers that comprise diverse subgroups of neoplasms. So far, despite the major epidemiologic concerns about the quality of care, limited data are available for patients with HMs. Thus, we created a novel measure—Quality of Care Index (QCI)—to appraise the quality of care in different populations.

**Methods:**

The Global Burden of Disease data from 1990 to 2017 applied in our study. We performed a principal component analysis on several secondary indices from the major primary indices, including incidence, prevalence, mortality, years of life lost, years lived with disability, and disability-adjusted life-years (DALYs) to create the QCI, which provides an overall score of 0–100 of the quality of cancer care. We estimated the QCI for each age group on different scales and constructed the gender disparity ratio to evaluate the gender disparity of care in HMs.

**Results:**

Globally, while the overall age-standardized incidence rate of HMs increased from 1990 to 2017, the age-standardized DALYs and death rates decreased during the same period. Across countries, in 2017, Iceland (100), New Zealand (100), Australia (99.9), and China (99.3) had the highest QCI scores for non-Hodgkin lymphoma, multiple myeloma, Hodgkin lymphoma, and leukemia. Conversely, Central African Republic (11.5 and 6.1), Eritrea (9.6), and Mongolia (5.4) had the lowest QCI scores for the mentioned malignancies respectively. Overall, the QCI score was positively associated with higher sociodemographic of nations, and was negatively associated with age advancing.

**Conclusions:**

The QCI provides a robust metric to evaluate the quality of care that empowers policymakers on their responsibility to allocate the resources effectively. We found that there is an association between development status and QCI and gender equity, indicating that instant policy attention is demanded to improve health-care access.

## Background

Hematologic malignancies (HMs) are a heterogeneous group of malignant disorders that are essential contributors in cancer global burden [1]. They are commonly classified by their four common subtypes: leukemia, Hodgkin lymphoma (HL), non-Hodgkin lymphoma (NHL), and multiple myeloma (MM) [2]. The diversity of leukemia mortality, incidence, origin, and pathogenesis relies on its subtype, which is generally classified as lymphoid and myeloid according to the world health organization (WHO) classification of tumors of hematopoietic and lymphoid tissue [2, 3]. In 2018, there were 407,000 incident cases of leukemia and 309,000 deaths [4]. Besides, in 2017, Hodgkin lymphoma, NHL, and MM accounted for 1.4, 7.0, and 2.3 million disability-adjusted life-years (DALYs) respectively [1]. Therefore, with regard to the increasing trend, more intensive attention should be paid to these patients.

The incidence of hematologic malignancies varies based on subtypes, age, gender, and socioeconomic state. For instance, between 1990 and 2017, a notable decrease in acute lymphocytic leukemia (ALL) and chronic myeloid leukemia (CML) incidence was discovered; however, the incidence rate of chronic lymphocytic leukemia (CLL) and acute myeloid leukemia (AML) has considerably raised in most countries [5]. The incidence cases of HL increased 38.6% from 1990 to 2017 [6]. Leukemia’s age-standardized incidence rate (ASIR) was higher in males compared to females [5]. Moreover, it has been revealed that the incidence of leukemia only has been increasing in people aged ≥ 70 years [5]. In terms of socioeconomic state, the highest incidence of leukemia occurred in the high-middle Socio-demographic Index (SDI) region [5]. As a result, proper prioritization of resources is crucial to reduce the undesired effects of increasing incidence of HMs.

Quality of care defines as supplying appropriate services for patients to access demanded health services. The American Society of Clinical Oncology (ASCO) has announced that there exists a profound divide based on race and ethnicity [7]. Moreover, yet, the disparity in the quality of cancer care is an essential obstacle of modern health care systems [7, 8]. The Global Burden of Disease (GBD) 2017 provided the incidence trends of leukemia; however, despite the major epidemiologic concerns about the quality of care and its components, comprehensive published data on the quality of care of HMs are scarce. Besides, the absence of a universal index to assess the quality of care in HMs is thought to be the principal problem in this journey.

In this article, considering the importance of the topic, we presented a new index of quality of care for HMs and used it to compare age groups, different regions, and genders in terms of HMs quality of care. Creating the index, would help to evaluate the hematologic malignancies’ quality of care in different geographical and age scales and enables effective policymaking and resource allocation.

## Materials and methods

### Overview and data resources

In this study, the GBD data form 1990 to 2017 presented in ‘GBD compare’ and IHME (Institute for Health Metrics and Evaluation) website, were retrieved. In the International Classification of Diseases, 10th Revision (ICD-10) system, HMs are documented under diagnosis codes, which are presented in Additional file 1: Table S1 [9, 10]. According to GBD codes for disease classification, HMs are registered as malignant neoplasms of blood cells under diagnosis codes B.1.25 for HL, B.1.26 for NHL, B.1.27 for MM, and B.1.28 for leukemia, which has subtypes including B.1.28.1 for ALL, B.1.28.2 for CLL, B.1.28.3 for AML, B.1.28.4 for CML, and B.1.28.5 for other leukemia [11]. This study is created based on GATHER guidelines [12].

### Quality of care index

We have produced four secondary indices from six primary indices in this study in order to assess the quality of care parameters. All these are indirect assessors of quality of care; which are: (1) years of life lost (YLLs) to years lived with disability (YLDs) ratio (2) disability-adjusted life year to prevalence ratio (3) mortality to incidence ratio (MIR) (4) prevalence to incidence ratio. Age-standardized original parameters were used for creation of these indices.

1 $$\mathrm{YLLs to YLDs Ratio}=\frac{\mathrm{ YLLs }}{\mathrm{ YLDs}}$$

2 $$\mathrm{DALYs to Prevalence Ratio}=\frac{\mathrm{ DALYs }}{\mathrm{ Prevalence}}$$

3 $$\mathrm{MIR}=\frac{\mathrm{ Mortality}}{\mathrm{ Incidence}}$$

4 $$\mathrm{Prevalence to Incidence Ratio}=\frac{\mathrm{ Prevalence }}{\mathrm{ Incidence}}$$

We utilized the principal component analysis (PCA) method to summarize these four indices into separate components. PCA is a multivariate statistical technique, which extracts a linear combination of different datasets as orthogonal components [13]. The first principal component, which is a linear combination of all variables, encloses the most information of these variables. The first component extracted from PCA was considered as the Quality of Care Index, which is mentioned as QCI in our study [14]. QCI scores were calculated and scaled into 0 to 100 range, with higher scores representing better quality of care. Moreover, similar indices like mortality to incidence ratio were utilized to assess the quality of care in different cancers [15, 16].

SDI is a scale of countries’ overall development, which is based on educational attainment, average income per person, and total fertility rate [17]. It is expressed on a scale of 0 to 1; values closer to zero indicating lower educational attainment, lower income per capita, and higher fertility rates observed across all GBD geographies. Besides, SDI is classified as low, low-middle, middle, high-middle, and high quintiles [17]. We measured QCI on SDI quintiles, as well as global scale, and WHO regions.

### Age and gender disparity

In this study, we categorized age as five years intervals, starting from one year old (1 to 4, 5 to 9, …, 90 to 95, and 95 plus). We utilized age-standardized measures as rates for 100,000 person-years in order to report and analyze the four secondary measures discussed earlier. We measured the QCI for each age group on different scales including SDI quintiles and global.

Gender disparity ratio (GDR), which is the QCI score for females divided by the QCI score for males, was created in order to appraise the gender disparity of care in HMs.

$$\mathrm{GDR}=\frac{\mathrm{ QCI for females}}{\mathrm{ QCI for males}}$$

Also, we further evaluated this ratio based on countries, SDI quintiles, and global scale. The least disparity of care between two sexes is represented as values close to one. Ratios lower or higher than one show difference in favor of one sex.

### Statistical analysis

The values of primary indices are presented with a 95% uncertainty interval (UI). Estimations and shifting trends were considered significant when UIs of two strata did not overlap. Also, we utilized the PCA method discussed above [13]. All the statistical analyses and plot depictions were performed by R statistical packages v3.6.1 (http://www.r-project.org/, RRID: SCR_001905) [18].

### QCI validity analysis

We have evaluated the correlation between the QCI and Healthcare Access and Quality Index (HAQI) by applying a mixed effect model of QCI as a dependent variable and inpatient health care utilization, outpatient health care utilization, cause-specific death, prevalence, and attributed death to all risk factor as independent variables and considering countries as random effects. Considering the issue that HL and leukemia are part of 32 causes that are amenable to health care, we calculated cause-specific correlation for these cancers [19]. The Pearson’s correlation coefficient between the predicted values with the HAQI was 0.85, 0.87, 0.85, and 0.81 for HL, NHL, MM, and leukemia, respectively, indicating that QCI and HAQI are essentially grasping similar components of health quality evaluation analysis. Besides, we constructed a Pearson’s correlation coefficient to assess the relation between predicted QCI and SDI. The results represented that all QCI values were notably positively correlated with SDI (Additional file 2: Table S2).

## Results

### Hodgkin lymphoma

Globally, there were 101,133 (95% UI 87,968 to 118,746) incident cases of Hodgkin lymphoma in 2017, which indicates a 38.6% increase in incidence between 1990 and 2017. Hodgkin lymphoma also accounted for an age-standardized death rate (ASDR) of 0.4 (0.4 to 0.5) per 100,000 person-years, indicating a decrease since 1990 (trend: − 45% [(5% UI − 36.4 to − 49.7]). In addition, DALYs showed a 43.6% (− 33.3% to − 49.8%) decrease during the same period (Table 1). In terms of SDI regions, high SDIs had the highest incidence rates; although, the mortality rate was the least in these areas. [More information about these indices and the trend of changes between 1990 and 2017 are presented in Additional file 3: Table S3- Sheet 1.]

**Table 1:** Primary indices’ rates of hematologic malignancies in 1990 and 2017 at the global level

Measure	Metric	Hodgkin lymphoma			Non-Hodgkin lymphoma			Multiple myeloma			Leukemia		
		1990 (95% UI)	2017 (95% UI)	Statistically Significant?	1990 (95% UI)	2017 (95% UI)	Statistically Significant?	1990 (95% UI)	2017 (95% UI)	Statistically Significant?	1990 (95% UI)	2017 (95% UI)	Statistically Significant?
Prevalence	Number	378,758 (286,249 to 414,343)	657,094 (568,931 to 780,837)	Yes	857,555 (828,438 to 895,762)	2,371,891 (2,324,951 to 2,418,127)	Yes	147,856 (132,552 to 171,190)	449,310 (414,568 to 520,850)	Yes	1,528,800 (1,272,203 to 1,797,889)	2,432,388 (2,190,332 to 2,591,602)	Yes
	Rate	7.2 (5.4 to 7.8)	8.3 (7.2 to 9.9)	No	18.9 (18.4 to 19.6)	29.7 (29.1 to 30.3)	Yes	3.5 (3.2 to 4.1)	5.5 (5.1 to 6.4)	Yes	30.2 (25.9 to 34.5)	32.4 (29 to 34.6)	No
Incidence	Number	72,937 (55,801 to 79,370)	101,133 (87,968 to 118,746)	Yes	206,863 (199,638 to 216,794)	487,964 (478,850 to 496,904)	Yes	65,487 (60,070 to 76,525)	152,746 (140,564 to 172,662)	Yes	354,471 (308,198 to 399,303)	518,485 (472,240 to 548,018)	Yes
	Rate	1.4 (1.1 to 1.6)	1.3 (1.1 to 1.5)	No	4.7 (4.6 to 4.9)	6.2 (6.1 to 6.3)	Yes	1.6 (1.5 to 1.9)	1.9 (1.8 to 2.2)	No	7.4 (6.6 to 8.2)	6.8 (6.1 to 7.2)	No
Death	Number	35,946 (27,329 to 39,412)	32,560 (27,644 to 38,086)	No	134,689 (129,673 to 142,079)	248,636 (243,475 to 253,064)	Yes	52,277 (48,480 to 61,265)	107,114 (98,521 to 118,911)	Yes	264,888 (234,391 to 292,591)	347,583 (317,256 to 364,877)	Yes
	Rate	0.8 (0.6 to 0.8)	0.4 (0.4 to 0.5)	Yes	3.2 (3.1 to 3.3)	3.2 (3.1 to 3.2)	No	1.3 (1.3 to 1.6)	1.4 (1.3 to 1.5)	No	5.8 (5.3 to 6.3)	4.5 (4.1 to 4.7)	Yes
YLLs	Number	1,625,133 (1,205,998 to 1,807,341)	1,327,631 (1,110,146 to 1,567,680)	No	4,497,053 (4,162,545 to 4,943,867)	6828,795 (6,611,841 to 7,019,991)	Yes	1,183,624 (1,095,061 to 1,349,698)	2,234,726 (2,091,390 to 2,493,151)	Yes	11,806,875 (9,724,465 to 13,876,452)	11,712,000 (10,531,416 to 12,523,323)	No
	Rate	30.9 (23.1 to 34)	17.1 (14.3 to 20.2)	Yes	92.9 (87.2 to 100.7)	86.8 (84 to 89.5)	No	28 (26 to 32.3)	27.7 (25.9 to 30.8)	No	221.9 (186.7 to 255.8)	153.4 (137.9 to 164.5)	Yes
YLDs	Number	32,336 (21,127 to 44,675)	50,534 (35,126 to 69,346)	No	76,792 (55,686 to 100,940)	193,118 (140,222 to 254,172)	Yes	32,531 (22,814 to 42,284)	90,764 (64,211 to 118,424)	Yes	164,720 (116,396 to 222,554)	263,348 (191,210 to 342,035)	No
	Rate	0.6 (0.4 to 0.9)	0.6 (0.4 to 0.9)	No	1.7 (1.3 to 2.3)	2.4 (1.8 to 3.2)	No	0.8 (0.5 to 1)	1.1 (0.8 to 1.5)	No	3.5 (2.5 to 4.6)	3.4 (2.5 to 4.5)	No
DALYs	Number	1,657,469 (1,228,330 to 1,843,223)	1,378,166 (1,155,059 to 1,624,392)	No	4,573,845 (4,236,062 to 5,018,937)	7,021,913 (6,794,727 to 7,232,477)	Yes	1,216,155 (1,127,669 to 1,388,918)	2,325,490 (2,176,068 to 2,606,756)	Yes	11,971,595 (9,868,922 to 14,047,583)	11,975,348 (10,749,148 to 12,793,575)	No
	Rate	31.5 (23.6 to 34.7)	17.8 (14.9 to 20.9)	Yes	94.6 (88.9 to 102.5)	89.3 (86.1 to 92.2)	No	28.8 (26.7 to 33.3)	28.8 (26.9 to 32.2)	No	225.4 (190.2 to 259.3)	156.8 (140.8 to 168.1)	Yes

Data are derived from GBD 2017. Both age-standardized and crude rates (with 95% uncertainty intervals) of primary indices are represented in the table. *DALYs* disability-adjusted life-years, *GBD* Global Burden of Disease, *YLDs* years lived with disability, *YLLs* years of life lost

**Fig. 1:**
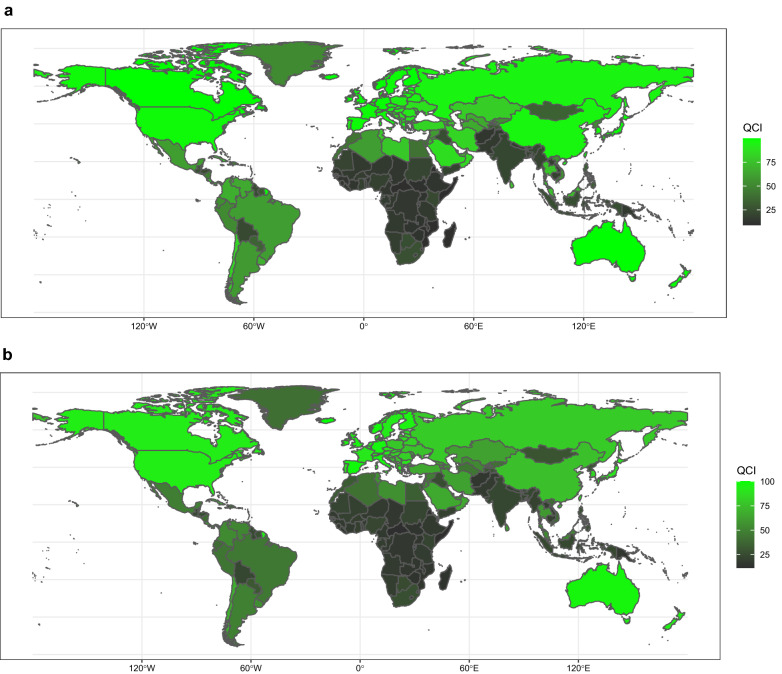

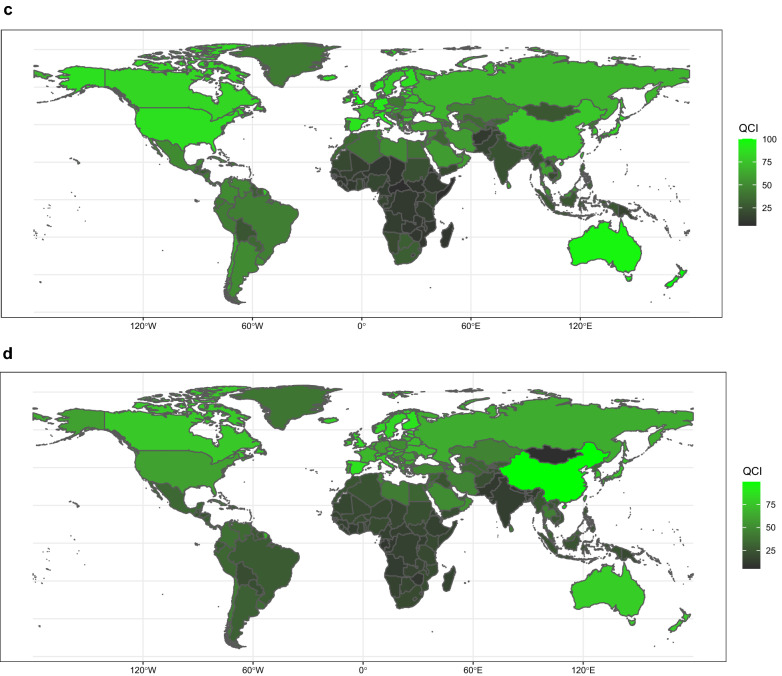
Age-standardized map of QCI scores, HL (**a**), NHL (**b**), MM **c**), and leukemia (**d**), 2017. The QCI scores are illustrated on a scale of 0–100, so that higher scores represent better quality of care. Countries and territories are pictured by their QCI scores on a color-based scale where grey represents the lowest scores and green represents the highest scores. *HL* Hodgkin lymphoma, *MM* multiple myeloma, *NHL* non-Hodgkin lymphoma, *QCI* Quality of Care Index

The overall age-standardized QCI score for Hodgkin lymphoma is 76.3. In 2017, QCI spanned from as high as 99.8 in Australia, Iceland, and Finland, to values as low as 9.6 in Eritrea, 9.8 in Somalia, and 10.2 in Burundi. From 1990 to 2017, 190 of 195 countries and territories had an increasing QCI, with the largest improvements in Maldives, followed by Turkey and Malaysia. QCI estimation in consideration of SDI quintiles spans from 98.2 for high SDI regions, to the score of 14.5 for low SDI areas (more details are displayed in Additional file 4: Table S4—Sheet1 and a global map of QCI distribution is indicated in Fig. 1a.)

**Fig. 2:**
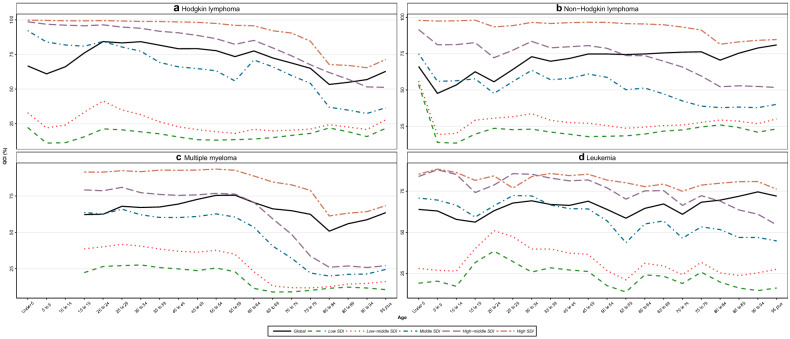
Age trend of QCI, HL (**a**), NHL (**b**), MM (**c**), and leukemia (**d**), 2017. The QCI scores are illustrated on a scale of 0–100, with 0 being the worst scores and 100 being the best scores. This figure demonstrates estimates for both sexes combined. Each line represents the association between QCI score and age in different regions including SDI quintile regions and global region. *HL* Hodgkin lymphoma, *MM* multiple myeloma, *NHL* non-Hodgkin lymphoma, *QCI* Quality of Care Index, *SDI* Socio-demographic Index

Investigation of QCI in different age groups in 2017, discloses global diversities in different ages. The QCI peaked at the ages of 20–24 (84.3), whereas the age group of 80–84 had the least QCI scores (53.3). There is a downward trend between ages 35 to 85, which gets to the lowest scores about age 85; however, QCI increases minutely after that. In different SDI regions, low and low-middle areas earned QCIs below global scores across all ages (Fig. 2a). The incidence of HL has higher rates in more advanced ages, except for ages of 30 to 45, followed by peaks at the age of 70 to 74. A similar pattern happens in mortality rate with the highest rate at the age of 85 to 89.

**Fig. 3:**
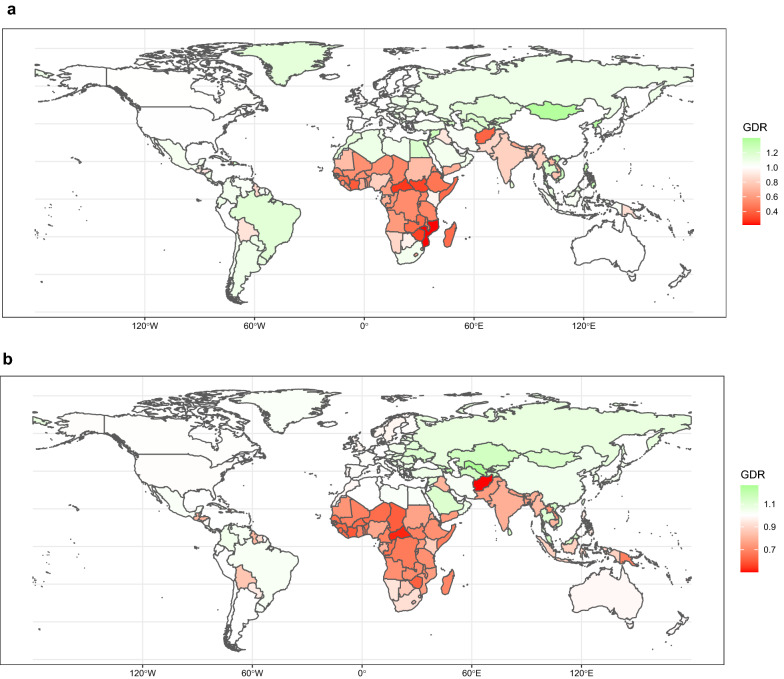

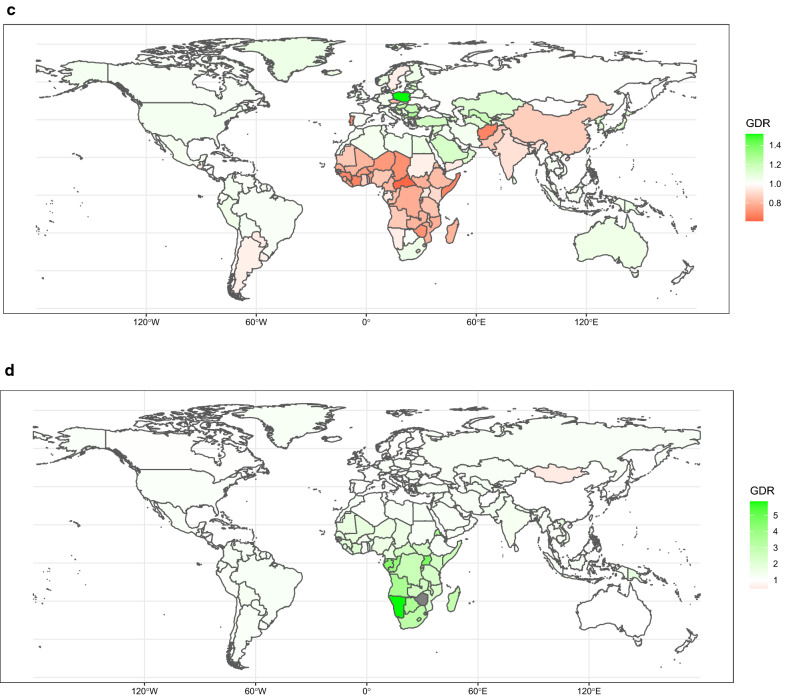
Age-standardized map of GDR, HL (**a**), NHL (**b**), MM (**c**), and leukemia (**d**), 2017. The GDR is QCI score for females divided by QCI score for males, so that higher scores represent better care in females and lower scores represent better care in men. This figure illustrates the GDR in different countries and territories. Countries and territories are pictured by their GDR on a color-based scale where white represents the absence of disparity. *GDR* gender disparity ratio, *HL* Hodgkin lymphoma, *MM* multiple myeloma, *NHL* non-Hodgkin lymphoma, *QCI* Quality of Care Index

The global map of the GDR of HL is presented in Fig. 3a. At the country level, American Samoa and Mongolia had the highest GDR, and Mozambique and Central African Republic had the lowest GDR, both demonstrating gender disparity. Among SDI regions, sex disparity in high SDI regions ranged from 1 to 1.2, which shows an almost equal condition of care between two sexes. In low SDI countries, lowest sex disparity is about 0.4 between ages 45 to 49. In 2017, the ASIR and ASDR were higher in men compared with women (more information about the GDR trend of different ages is indicated in Additional file 8: Figure S4A).

### Non-Hodgkin lymphoma

In 2017, there were 487,964 (95% UI 478,850 to 496,904) incident cases worldwide, with an age-standardized incident rate of 6.2 (6.1 to 6.3) per 100,000 person-years. The ASIR showed an increase of 30.1% (23.9–35.3) from 1990 to 2017. In addition, high SDI regions earned the greatest ASIR compared with other regions. Non-Hodgkin lymphoma caused 248,636 (243,475 to 253,064) deaths, with an age-standardized rate of 3.2 (3.1 to 3.2) per 100,000 person-years, which was stable over the past 28 years. DALYs for non-Hodgkin lymphoma are estimated 7.0 million (6.8–7.2) years with a rate of 89.3 (86.1 to 92.2), which showed a decrease of 5.6% (–15 to –2.2) compared with its rate in 1990 (Table 1; Additional file 3: Table S3- Sheet 2).

The overall age-standardized QCI for non-Hodgkin lymphoma equals 70.2. Among different countries, Iceland (100), Luxembourg (98.3), and Spain (98.2) reached the highest QCI in 2017. By contrast, Central African Republic (11.5), Somalia (12.6), and Eritrea (12.8) had the lowest QCI (Fig. 1b). From 1990 to 2017, QCI increased in 193 of 195 countries, with China, Lebanon, and South Korea recording among the largest gains by 2017. By SDI quintiles, QCI tended to be raised in high SDI regions relative to lower SDIs (Additional file 4: Table S4- Sheet 2).

Given the differential trends by age, age groups above 95 years had the highest QCI score, while the age group between 5 to 9 had the lowest QCI score. The QCI score fluctuates between age 10 and 95; however, it generally has an upward trend. Among different SDIs, the high SDI is above global quality across all ages, whereas low and low-middle SDI regions are below global scores across all ages except for the age group of under five years in middle SDI regions (Fig. 2b). The ASIR for non-Hodgkin lymphoma has higher values in higher age groups, which ascends sharply after the age of about 40 years.

Analysis of GDR in different regions represents global diversity, with the highest ratio in Uzbekistan and Azerbaijan, which shows better care in women, and the lowest ratio in Afghanistan and Central African Republic, favoring better care in men (Fig. 3b). Besides, low SDI regions have the lowest GDR, which is 0.4 in the age group 20 to 24. Evaluation of age-standardized incidence and death rates by sex showed that the highest ASIR and ASDR were in men. Also, the association between GDR and age is presented in Additional file 8: Figure S4B.

### Multiple myeloma

In 2017, there were 152,746 (140,564 to 172,662) incident cases of MM, with an ASIR of 1.9 (1.8 to 2.2) per 100,000 person-years. By SDI level, high SDI regions had the greatest ASIR. Multiple myeloma was responsible for 107,114 (98,521 to 118,911) deaths worldwide, with an ASDR of 1.4 per 100,000 persons (95%UI, 1.3–1.5). Also, MM accounted for 2.3 million (2.2–2.6) DALYs at the global level in 2017. From 1990 to 2017, ASIR of MM increased by 16.9% (8.3–22.2); however, death rate was stable during the same period (Table 1; Additional file 3: Table S3—Sheet 3).

At the global level, the average age-standardized QCI score is estimated about 68.6. Countries with the highest QCI scores were New Zealand (100), Australasia (96.8), and United Kingdom (96.8). Conversely, Central African Republic (6.1), Somalia (7.1), and Burundi (9.5) earned the lowest scores (Fig. 1c). The percent change in QCI score from 1990 to 2017 differed substantially between countries, with Equatorial Guinea and Bangladesh showing the largest increases. In terms of SDI, the QCI score of multiple myeloma showed greatest numbers in regions with higher SDIs (Additional file 4: Table S4- Sheet3).

The QCI score followed distinct age patterns in 2017, with age groups between 55 and 59 had the highest scores and age groups between 80 and 84 had the lowest scores. The QCI score increased in a non-linear manner up to the age of 59, and decreased after that except for age groups older than 85 years. Middle, low-middle, and low SDI areas are below global scores across all ages; while, high SDI regions are above global scores except for age groups older than 80 years (Fig. 2c). The global incidence of MM has higher rates in higher age groups, with the highest rates occurring at ages 90 to 94, which have a relatively similar pattern with death rates.

The global trend of GDR in 2017 showed scores lower than one across all ages, with lowest scores between 80 and 84 years and highest scores between 55 and 59 years. Moreover, analysis of GDR in different SDI regions revealed than all SDI regions had scores lower than one across all age groups, except for high SDI regions in age groups lower than 75 years, which proves an unequal condition of care in two sex, favoring better care in men (Additional file 8: Figure S4C). By country, Poland and Bulgaria had the highest ratio; while, Central African Republic and Cote d'Ivoire had the lowest ratio (Fig. 3c). Moreover, the highest ASIR and death rates were in men.

### Leukemia

Globally, despite the increases in newly diagnosed leukemia cases from 354.5 to 518 thousand between1990 and 2017, the ASIR decreased by 8.8% (–20.4 to –1.1) during the same period, which particularly came from the decrease in ASIR of ALL and CML. In 2017, Leukemia contributed to 347,583 (317,256 to 364,877) deaths with an age-standardized rate of 4.5 (4.1 to 4.7) per 100,000 person-years, which showed a 22.5% decrease compared with the same rate in 1990. In addition, leukemia resulted in about 12.0 million (10.7–12.1) DALYs in 2017, with an age-standardized rate of 156.8 (140.8 to 168.1) per 100,000 person-years. Also, more information about primary indices of leukemia’ subtypes is presented in Additional file 3: Table S3- Sheets 4–9.

The average QCI score for leukemia is 57.5 worldwide. The highest QCI scores by country were estimated in China (99.3), Finland (87.9), and Spain (86.9), and the lowest in Mongolia (5.4), Fiji (6.6), and Gabon (9.5) (Fig. 1d). Between 1990 and 2017, the percent change in the QCI score was highest in Mongolia and South Korea. In 1990, the highest QCI score was in high SDI regions (56.4), while in 2017, high-middle SDI regions acquired the highest score, which showed a significant increase in QCI scores for high-middle SDI areas during the study period. More details about subtypes of leukemia are presented in Additional file 4: Table S4- Sheets 4–9, and Additional file 5: Figure S1A-E.

The overall QCI score conveys marked differences between age groups, with the highest scores in the age group from 90 to 94 and lowest scores between ages 15 and 19. The QCI score trend was bimodal for both women and men, with the first peak occurring at ages 30–34, and a second peak and highest scores at ages 90–94 (Fig. 2d). At the SDI level, low and low-middle areas were below global scores across all ages. On the contrary, high and high-middle areas were above global scores across almost all ages. The association between the QCI scores of leukemia’s subtypes and age is displayed in Additional file 6: Figure S2A-E. The ASIR pattern of leukemia showed a similar trend with death rates, both have typically higher rates at higher ages except for incident rate at the age group of lower than 5 years.

Considering the GDR variety between different countries, we see the highest GDR in Namibia and Uganda, and the lowest GDR in Mongolia and Sweden (Fig. 3d, and Additional file 7: Figure S3A-E). Since the GDR of Fiji, Zimbabwe, and Swaziland were classified as outliers, we removed them from further analyses. By SDI regions, GDR ranges from 0.9 to 1.1 in high SDI regions, indicating equal care between two sexes. We also see the highest ASIR and ASDR in men compared with women. In addition, the association between GDR of leukemia’s subtypes and age, globally, and by SDI quintile regions is illustrated in Additional file 8: Figure S4D-D5.

## Discussion

Drawing from GBD 2017, this is the first study to quantify the personal health-care quality as a novel measure called Quality of Care Index. We found that despite the downward trend in DALYs, HMs make up a considerable proportion of the burden of neoplasms, with increases in ASIR for all HMs since 1990 with the exception of leukemia, ALL, and CML. Our analysis showed that the quality of care in developed areas and high SDI regions is notably higher. Overall, ASIR and ASDR increased and QCI scores decreased with advancing age. Besides, the ASIR and ASDR of HMs were remarkably higher in men compared with women. Moreover, many geographical locations displayed imperative gender disparities in regard to access to health care and quality care.

Hematologic malignancies generally originate in different ways as follows: Myeloid neoplasms including AML and CML arise from mutations in bone marrow progenitor cells that commonly develop into granulocytes or erythrocytes [20]. On the other hand, lymphoid neoplasms such as ALL and CLL result from B or T cell progenitors or mature B or T lymphocytes [20]. Lymphomas are a compound group of malignancies of T cells, B cells, and natural killer (NK) cells that commonly emanate in the lymph nodes [20]. MM originates from the proliferation of a clone of malignant plasma cells [21]. So far, the cause of HMs has been disputed; however, several genetic and environmental risk factors including neurofibromatosis and Down’s syndrome, which result in leukemia, and ionizing radiation, as an important cause for childhood ALL, have been identified [22, 23]. Besides, smoking, high body mass index, and occupational exposures to formaldehyde and benzene have been considered as the essential contributors to AML and ALL-related death and DALY [24, 25].

Analysis of primary epidemiologic indices of HMs demonstrates impressive alteration from 1990 to 2017, with increases in ASIR for all hematologic malignancies with the exception of leukemia, ALL, and CML, which may have been driven by new screening strategies and increasing risk factors [4, 26, 27]. The decreases in the death rate of almost all of the HMs could be explained by treatment improvement and increased access to cancer care [28]. Overall, our study highlights that the ASIR of all hematologic malignancies increased, along with increases in SDI, which is in line with those of previous studies [5, 6, 29]. Consistent with this concept, Yi and colleagues [24] studied the global burden and attributable risk factors of AML and found that ASIR values were positively correlated with SDI. The difference in incidence could be postulated to be driven by better screening programs, better diagnostic abilities, and population aging in higher SDI regions compared with lower SDI countries [30, 31].

The QCI provides a robust metric for appraising the quality of care in different populations. This potency is crucial since attaining the global health coverage is an essential purpose for all countries, and thus this comparable index enables policy-makers to allocate health resources. In a cohort study, 113 patients with hematologic malignancies were compared to 703 patients with solid tumors base on the quality of end-of-life care. It has been concluded that patients with hematological malignancies had higher intensive care unit admissions, emergency room visits, malignancy treatment, and death in the last week of life compared with other patients, which highlights the important role of programs enhancing the quality of care in patients with HMs [32]. Of note, there are several obstacles to reach a sufficient quality of care. It has been revealed that insurance policies may be an important barrier to reach an ideal quality of care [8]. In addition, Burg and colleagues designed a study on the Association of Oncology Social Work members and classified the barriers to accessing quality healthcare for cancer patients as individual barriers, environmental/social barriers, and health system barriers. They concluded that the inability to pay treatment and lack of health insurance were of the most important issues, which put an emphasis on the issue that supplying adequate insurance coverage can lead to a better quality of care [33].

Accessing hematologic malignancies pattern, the elevated QCI scores in higher SDI regions are noteworthy, which shows that increased efforts are demanded to overcome these health inequalities. Also, we conducted more detailed inspections of countries with higher QCI scores, and found that generally Nordic countries including Iceland and Finland, Australia, New Zealand, and China (only for leukemia) had the highest QCI scores. Evaluation of these results showed that there are essential factors that contributed to these achievements. First, most of these countries have HMs management guidelines and screening programs; for instance, the European Society for Medical Oncology (ESMO) guideline for hematologic malignancies provides a valuable approach to the management of these cancers [34]. Second, the development of insurance coverage and considerable investment in the health system since 2003 in China, have contributed to a notable increase in cancer survival between 2003 and 2015, which represents an overall progression in the quality of cancer care [35, 36]. Third, the establishment of efficient cancer registries has led to better prediction and thus higher quality of care. For instance, it has been revealed that the Association of the Nordic Cancer Registries (ANCR) illustrates accurate registered data, resulting in achieving better quality of care [37]. Besides, the National Blood Cancer Registry (NBCR) in Australia and New Zealand intends to improve the outcome of patients with HMs [38].

On the other hand, hematologic malignancies are an essential health problem in Sub-Saharan Africa. Countries located in this region including Eritrea, Somalia, Burundi, and Central African Republic had the lowest QCI score in our analysis. Possible explanations for this issue are lack of awareness, deficiency of financial resources, lack of cancer registration centers, as well as resources for diagnosing HMs, and treatment and palliative services scarcity [39, 40]. To achieve the adequate quality of cancer care, there will be legitimate debate about the achievable and implantable strategies [39]. Although, we believe that by adjusting and reshaping the infrastructures, the existing gap in the quality of HMs care in Africa could be narrowed. In this regard, particular recommendations are as follows. First, to enhance the population perception, integration of cancer prevention projects into educational foundations should be noticed [39]. Second, due to the resource constraints and deficiency of financial resources, developing cancer control measures regarding the cost-effective analysis, as well as increasing budgetary allocation to health care insurance could be the essential future priorities [40, 41]. Third, lack of cancer registration centers is of utmost importance given that the underreporting of cancer trends affects cancer monitoring, prevention, and policymaking [40, 42]. Across African countries, the implementation of Cancer Care Center (CCC) in Northern Tanzania has played a pivotal role in enhancing the quality of HMs care [42]. Besides, the International Agency for Research on Cancer (IARC) has supported the development of cancer registries through African countries [39]. However, the need for additional cancer registry centers has never been timelier, since the cancer incidence trend continues to increase in this region [4, 39].

Without accurate diagnosis of HMs, neither prevention strategies nor practicable management can be instituted [43]. In support of this concept, the International Network for Cancer Treatment and Research (INCTR) has ushered African centers with approaches exclusively designed for the prevailing infrastructure in these regions [43]. However, limited diagnostic hematopathology tests and pathologists scarcity are still considered as the essential barriers to access the aspirational goals [40]. Hence, the collaboration of African countries with their European/North American counterparts could help to improve diagnostic standards through both direct and indirect technology transfer, deliberations, and capacity building [43]. Last but not least, African governments’ inability to provide adequate quantities of HMs treatments imposes a great concern on public health [44]. Lack of infrastructures to support allogeneic hematopoietic stem cell transplantation (HSCT) and targeted molecular therapies scarcity are among the major barriers to achieve a standard treatment protocol [40]. In sub-Saharan Africa, the cornerstone of successful human immunodeficiency virus (HIV) management has resulted from the agreements with manufacturers to supply HIV medicines at low cost [40, 45]. Hence, there is now the same opportunity to substantially improve the HMs management in the years to come.

With regard to the shifting trend in epidemiological profiles, we see most countries have improved in their QCI scores since 1990, with the most improvement recorded for countries including China, Maldives, and Turkey. For instance, the health transformation program in Turkey contributed to an increase in health services [46]. Overall, these trends could mirror the role of health insurance development and government health policies in improving the quality of care [46, 47]. Comparing trends in epidemiological studies is a common feature. Our findings in term of the regional pattern of QCI scores nearly is consistent with that of the HAQ study in 2016, which reported the highest scores in Iceland and the lowest in Central African Republic [19].

Evaluation of HMs QCI in different age groups indicates the overall lower quality of care in older ages for MM and Hodgkin lymphoma, which could be a result of the multi-morbidity presence, accumulation of damaged deoxyribonucleic acid (DNA), diminished function of the immune system, and rapid population aging tendency [48, 49]. By contrast, NHL and leukemia had higher QCI scores in older ages. There are some possible reasons for this trend in older ages. First, due to the presence of different comorbidities in elderly patients, which can contribute to death, younger patients commonly are exposed to the disease for a longer period time, resulting in lower quality of care in these patients [50]. Second, GBD data are sub-optimally unreliable in age groups older than 80 years, which may affect some of the results in these age groups. The ASIR and death rate of all HMs increased steadily with advancing age except for ALL and Hodgkin lymphoma, which displayed bimodal patterns. A two-hit model has been suggested for the childhood peak of ALL, whereby the first hit happens due to genetic defects during the prenatal time, and the second occurs as a result of environmental factors during the postnatal time [51]. This finding is nearly consistent with that of Yi M, et al. [25] who analyzed the global burden and trend of ALL and reported two peaks for this cancer: the first peak in children (under five years old) and the other peak in the elderly. Also, the bimodal age pattern of Hodgkin lymphoma has been reported to be due to the co-infections with this cancer in young adults [52].

Interpretation of global HMs GDR shows a remarkable diversity across different gender groups. In contrast with low SDI regions, there is equal care for both sexes in high SDI areas, indicating an equitable distribution of health resources for both sexes in SDI regions. Besides, the GDR showed better care in men across all ages in multiple myeloma. These findings could be explained by the following reasons: (1) compared with developed areas, women with cancer have a lower priority in developing regions, which demonstrates inequality in cancer care. (2) It has been revealed that women generally have higher pain perception compared with men, which could inform health-care clinicians to make decisions in order to improve women’s quality of care [53, 54]. In addition, for all hematologic cancers, standardized mortality and incidence rates have been detected to be higher in men. In support of this issue, several studies have shed some light on the higher possibility of HMs development in males compared with females [24, 25]. This phenomenon could be the result of the following reasons: first, more exposure to occupational and environmental risk factors is detected in men [55, 56]. Second, the higher rates of HIV infection in men may be responsible for the higher NHL incidence among them [57, 58]. Third, the greater prevalence of alcohol consumption and smoking in men can lead to a higher incidence of HMs compared with women [59, 60].

## Strengths and limitations

The key strengths of our study are as follows: first, this is the first study that addresses the quality of care for HMs globally. Furthermore, the estimate of QCI has been validated by HAQI, providing a robust metric to understand the untapped potential for quality of cancer care improvement. In addition, institution of this index in HMs would help to understand better controversies on different studies, the real burden of HMs, and its impact on health services. Last but not least, the QCI could represent the inequalities in HMs across different ages, genders, and developmental status, which enables better understanding of priorities to enhance the quality of cancer care worldwide. We would like to emphasize that our study has some limitations. First, owing to the limitations of IHME-GBD datasets in the data registry of countries, our results might be affected in some areas due to modeling that has been used for addressing the scarcity of data. Second, our study does not cover the ethnic and racial disparities evaluation since the absence of this information in the GBD dataset. Third, some subtypes of leukemia are not evaluated separately in the GBD and are reported under the aggregated cause “other leukemia”. Fourth, the GBD data look sub-optimally reliable in age groups older than 80 years.

## Conclusions

The QCI score enables comparable evaluation of health care quality across different regions, and provides valuable information to allocate health resources. So far, notable geographical inequalities exist across different regions, indicating that there is a direct association between development status and QCI and gender equity. Therefore, attention should be sought in order to improve access to health-care goods in areas being left behind.

## Supplementary Information

**Additional file 1: Table S1.** International Classification of Disease, 10th Revision system, codes mapped for hematologic malignancies. Mortality and morbidity codes of the International Classification of Disease, 10th Revision (ICD-10) system, for HMs are illustrated in the table. Abbreviations: ALL = acute lymphocytic leukemia. AML = acute myeloid leukemia. CLL = chronic lymphocytic leukemia. CML = chronic myeloid leukemia. HMs = hematologic malignancies.

**Additional file 2: Table S2.** Pearson’s correlation coefficient between the predicted QCI of hematologic malignancies and HAQI and SDI values. The Pearson’s correlation coefficient between the predicted QCI of hematologic malignancies and HAQI and SDI values is evaluated by applying a mixed effect regression model of QCI as a dependent variable and inpatient health care utilization, outpatient health care utilization, cause-specific death, prevalence, and attributed death to all risk factor as independent variables and considering countries as random effects. Abbreviations: ALL = acute lymphocytic leukemia. AML = acute myeloid leukemia. CLL = chronic lymphocytic leukemia. CML = chronic myeloid leukemia. HAQI = Healthcare Access and Quality Index. QCI = Quality of Care Index. SDI = Socio-demographic Index.

**Additional file 3: Table S3.** Age-standardized and crude rates of primary indices (and 95% uncertainty intervals) by global, socio-demographic index quintile regions, WHO regions, and 195 countries or territories. Data are taken from GBD 2017. All primary indices including prevalence, incidence, deaths, YLLs, YLDs, and DALYs are illustrated for HL, NHL, MM, leukemia, and subtypes of leukemia in 1990 and 2017. Age-standardized rates are reported per 100,000 person-years. Abbreviations: ALL = acute lymphocytic leukemia. AML = acute myeloid leukemia. CLL = chronic lymphocytic leukemia. CML = chronic myeloid leukemia. DALYs = disability-Adjusted life-years. GBD = Global Burden of Disease. HL = Hodgkin lymphoma. MM = multiple myeloma. NHL = non-Hodgkin lymphoma. SDI = Socio-demographic Index. WHO = World Health Organization. YLDs = years lived with disability. YLLs = years of life lost.

**Additional file 4: Table S4.** Quality of Care Index and gender disparity ratio of hematologic malignancies by global, socio-demographic index quintile regions, WHO regions, and 195 countries or territories. Data are derived from GBD 2017. QCI scores are represented for HL, NHL, MM, leukemia, and subtypes of leukemia in females, males, and both sexes in 1990 and 2017. Abbreviations: ALL = acute lymphocytic leukemia. AML = acute myeloid leukemia. CLL = chronic lymphocytic leukemia. CML = chronic myeloid leukemia. DALYs = disability-adjusted life-years. GBD = Global Burden of Disease. HL = Hodgkin lymphoma. MM = multiple myeloma. NHL = non-Hodgkin lymphoma. QCI = Quality of Care Index. SDI = Socio-demographic Index. WHO = World Health Organization. YLDs = years lived with disability. YLLs = years of life lost.

**Additional file 5: Figure S1.** Age-standardized map of QCI scores at the national level, for AML (A), ALL (B), CML (C), CLL (D), and other leukemia (E), 2017. The QCI scores are illustrated on a scale of 0–100, so that higher scores represent better quality of care. Countries and territories are pictured by their QCI scores on a color-based scale where grey represents the lowest scores and green represents the highest scores. Abbreviations: ALL = acute lymphocytic leukemia. AML = acute myeloid leukemia. CLL = chronic lymphocytic leukemia. CML = chronic myeloid leukemia. QCI = Quality of Care Index.

**Additional file 6: Figure S2.** Range of QCI scores in different ages for AML (A), ALL (B), CML (C), CLL (D), and other leukemia (E), globally and by SDI quintile, 2017. The QCI scores are illustrated on a scale of 0–100, with 0 being the worst scores and 100 being the best scores. This figure demonstrates estimates for both sexes combined. Each line shows the association between QCI score and age in different areas including SDI quintile regions and global region. Abbreviations: ALL = acute lymphocytic leukemia. AML = acute myeloid leukemia. CLL = chronic lymphocytic leukemia. CML = chronic myeloid leukemia. QCI = Quality of Care Index. SDI = Socio-demographic Index.

**Additional file 7: Figure S3.** Age-standardized global map of gender disparity ratio, AML (A), ALL (B), CML (C), CLL (D), and other leukemia (E), 2017. The GDR is QCI score for females divided by QCI score for males, so that higher scores represent better care in females and lower scores represent better care in men. This figure illustrates the GDR in different countries and territories. Countries and territories are pictured by their GDR on a color-based scale where white represents the absence of disparity. Abbreviations: ALL = acute lymphocytic leukemia. AML = acute myeloid leukemia. CLL = chronic lymphocytic leukemia. CML = chronic myeloid leukemia. GDR = gender disparity ratio. QCI = Quality of Care Index.

**Additional file 8: Figure S4.** The association between gender disparity ratio and age for all hematologic malignancies globally and by SDI quintile regions in 2017. This figure demonstrates estimates for both sexes combined. Each line shows the association between GDR and age in different areas including SDI quintile regions and global region. Range of GDR is represented in nine figures for different subtypes of hematologic malignancies including HL (A), NHL (B), MM (C), leukemia (D), ALL (D1), CLL (D2), AML (D3), CML (D4), and other leukemia (D5). Abbreviations: ALL = acute lymphocytic leukemia. AML = acute myeloid leukemia. CLL = chronic lymphocytic leukemia. CML = chronic myeloid leukemia. GDR = gender disparity ratio. HL = Hodgkin lymphoma. MM = multiple myeloma. NHL = non-Hodgkin lymphoma. SDI = Socio-demographic Index.

## Data Availability

The datasets generated and/or analyzed during the current study are available in the GBD repository, [https://gbd2017.healthdata.org/gbd-search/].

## Abbreviations

ALL: Acute lymphocytic leukemia
AML: Acute myeloid leukemia.
ANCR: Association of the Nordic Cancer Registries
ASCO: American Society of Clinical Oncology
ASDR: Age-standardized death rate
ASIR: Age-standardized incidence rate
CCC: Cancer Care Center
CLL: Chronic lymphocytic leukemia
CML: Chronic myeloid leukemia
DALYs: Disability-adjusted life-years
DNA: Deoxyribonucleic acid
ESMO: European Society for Medical Oncology
GBD: Global Burden of Disease
GDR: Gender disparity ratio
HAQI: Healthcare Access and Quality Index
HIV: Human immunodeficiency virus
HL: Hodgkin lymphoma
HMs: Hematologic malignancies
HSCT: Hematopoietic stem cell transplantation
IARC: International Agency for Research on Cancer
ICD-10: International Classification of Diseases, 10th Revision
IHME: Institute for Health Metrics and Evaluation
INCTR: International Network for Cancer Treatment and Research
MIR: Mortality to incidence ratio
MM: Multiple myeloma
NBCR: National Blood Cancer Registry
NHL: Non-Hodgkin lymphoma
PCA: Principal component analysis
QCI: Quality of Care Index
SDI: Socio-demographic Index
SUP: Supplementary
UI: Uncertainty interval
WHO: World Health Organization
YLDs: Years lived with disability
YLLs: Years of life lost
