# Supplementary material for: A global, regional, and national survey on burden and Quality of Care Index (QCI) of hematologic malignancies; global burden of disease systematic analysis 1990–2017
Source: Exp Hematol Oncol. 2021 Feb 8;10:11. doi: 10.1186/s40164-021-00198-2 (PMC7869509; doi:10.1186/s40164-021-00198-2)
Supplement: Supplementary file 1 — Additional file 1: Table S1. International Classification of Disease, 10th Revision system, codes mapped for hematologic malignancies. Mortality and morbidity codes of the International Classification of Disease, 10th Revision (ICD-10) system, for HMs are illustrated in the table. Abbreviations: ALL = acute lymphocytic leukemia. AML = acute myeloid leukemia. CLL = chronic lymphocytic leukemia. CML = chronic myeloid leukemia. HMs = hematologic malignancies. [file 40164_2021_198_MOESM1_ESM.docx]

**Table S1.** International Classification of Disease, 10th Revision system, codes mapped for hematologic malignancies.

|  | Mortality | Morbidity |
| --- | --- | --- |
| Hodgkin lymphoma | C81-C81.9 | C81-C81.49, C81.7-C81.79, C81.9-  C81.99, Z85.71-Z85.72 |
| Non-Hodgkin lymphoma | C82-C86.6, C96-C96.9 | C82-C85.29, C85.7-C86.6, C96-C96.9 |
| Multiple myeloma | C88-C90.9 | C88-C90.32 |
| Leukemia | C91-C95.9 | C91-C93.7, C93.9-C95.2, C95.7-C95.92,  Z80.6, Z85.6 |
| ALL | C91.0 | C91.0-C91.02 |
| CLL | C91.1 | C91.1-C91.12 |
| AML | C92.0, C92.3-C92.6, C93.0, C94.0, C94.2, C94.4-C94.5 | C92.0-C92.02, C92.3-C92.62, C93.0-  C93.02, C94.0-C94.02, C94.2-C94.22, |
| CML | C92.1 | C92.1-C92.12 |
| Other leukemia | C91.2-C91.9, C92.2, C92.7-C92.9, C93.1-C93.9, C94.1, C94.3, C94.6-  C95.9 | - |

Mortality and morbidity codes of International Classification of Disease, 10th Revision (ICD-10) system, for HMs are illustrated in the table. Abbreviations: ALL= acute lymphocytic leukemia. AML= acute myeloid leukemia. CLL= chronic lymphocytic leukemia. CML= chronic myeloid leukemia. HMs= hematologic malignancies.
