# Supplementary material for: A global, regional, and national survey on burden and Quality of Care Index (QCI) of hematologic malignancies; global burden of disease systematic analysis 1990–2017
Source: Exp Hematol Oncol. 2021 Feb 8;10:11. doi: 10.1186/s40164-021-00198-2 (PMC7869509; doi:10.1186/s40164-021-00198-2)
Supplement: Supplementary file 2 — Additional file 2: Table S2. Pearson’s correlation coefficient between the predicted QCI of hematologic malignancies and HAQI and SDI values. The Pearson’s correlation coefficient between the predicted QCI of hematologic malignancies and HAQI and SDI values is evaluated by applying a mixed effect regression model of QCI as a dependent variable and inpatient health care utilization, outpatient health care utilization, cause-specific death, prevalence, and attributed death to all risk factor as independent variables and considering countries as random effects. Abbreviations: ALL = acute lymphocytic leukemia. AML = acute myeloid leukemia. CLL = chronic lymphocytic leukemia. CML = chronic myeloid leukemia. HAQI = Healthcare Access and Quality Index. QCI = Quality of Care Index. SDI = Socio-demographic Index. [file 40164_2021_198_MOESM2_ESM.docx]

**Table S2.** Pearson’s correlation coefficient between the predicted QCI of hematologic malignancies and HAQI and SDI values.

| Cause | HAQI | SDI |
| --- | --- | --- |
| Hodgkin lymphoma | 0.85 | 0.79 |
| Non-Hodgkin lymphoma | 0.87 | 0.83 |
| Multiple myeloma | 0.85 | 0.83 |
| Leukemia | 0.81 | 0.70 |
| ALL | 0.81 | 0.69 |
| CLL | 0.79 | 0.72 |
| AML | 0.61 | 0.67 |
| CML | 0.70 | 0.72 |
| Other leukemia | 0.45 | 0.58 |

The Pearson’s correlation coefficient between the predicted QCI of hematologic malignancies and HAQI and SDI values is evaluated by applying a mixed effect regression model of QCI as a dependent variable and inpatient health care utilization, outpatient health care utilization, cause-specific death, prevalence, and attributed death to all risk factor as independent variables and considering countries as random effects. Abbreviations: ALL= acute lymphocytic leukemia. AML= acute myeloid leukemia. CLL= chronic lymphocytic leukemia. CML= chronic myeloid leukemia. HAQI= Healthcare Access and Quality Index. QCI= Quality of Care Index. SDI= Socio-demographic Index.
