# Supplementary figures and images for: A global, regional, and national survey on burden and Quality of Care Index (QCI) of hematologic malignancies; global burden of disease systematic analysis 1990–2017
Source: Exp Hematol Oncol. 2021 Feb 8;10:11. doi: 10.1186/s40164-021-00198-2 (PMC7869509; doi:10.1186/s40164-021-00198-2)

A

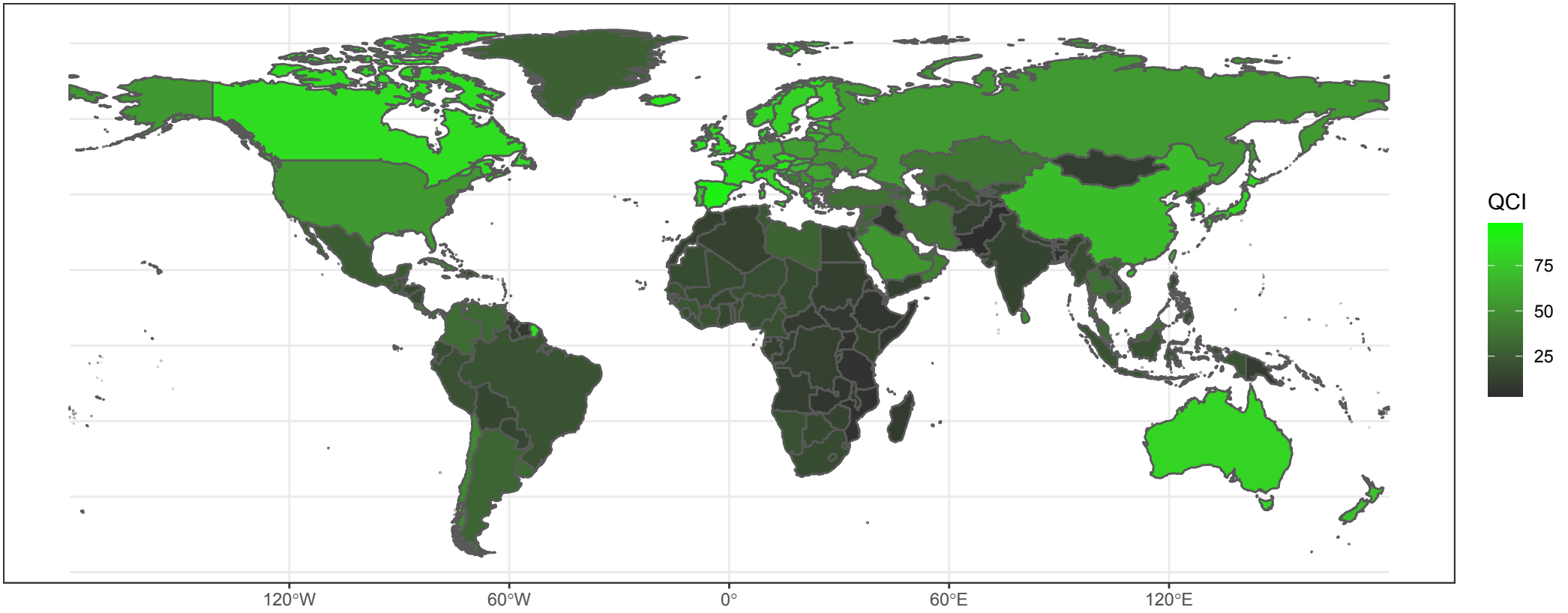

B

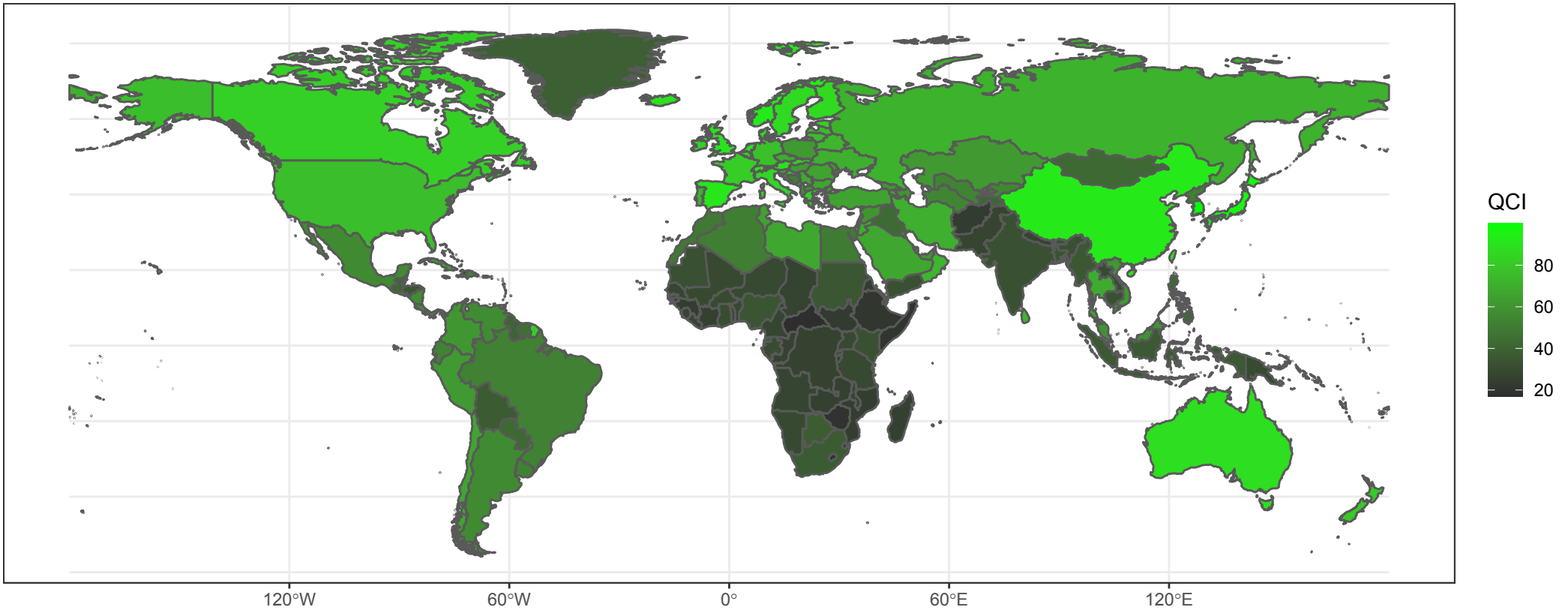

C

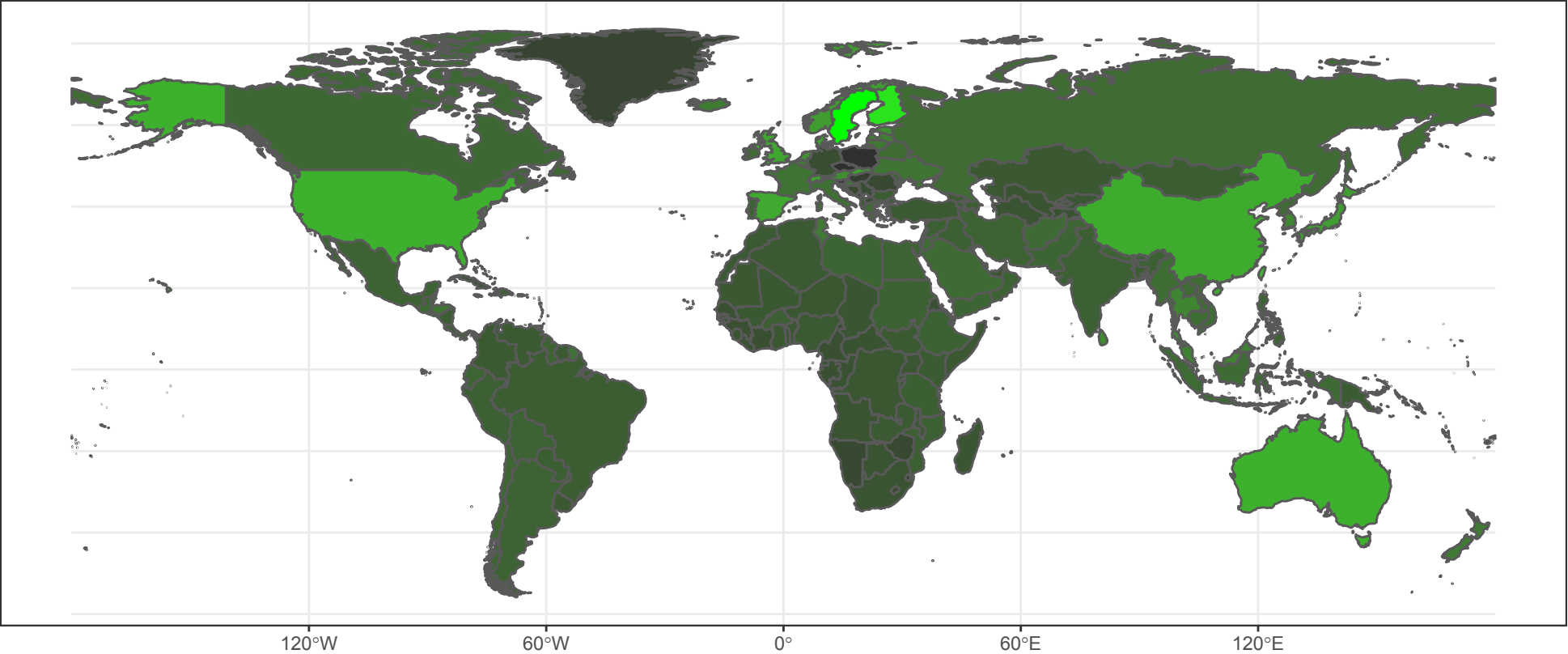

D

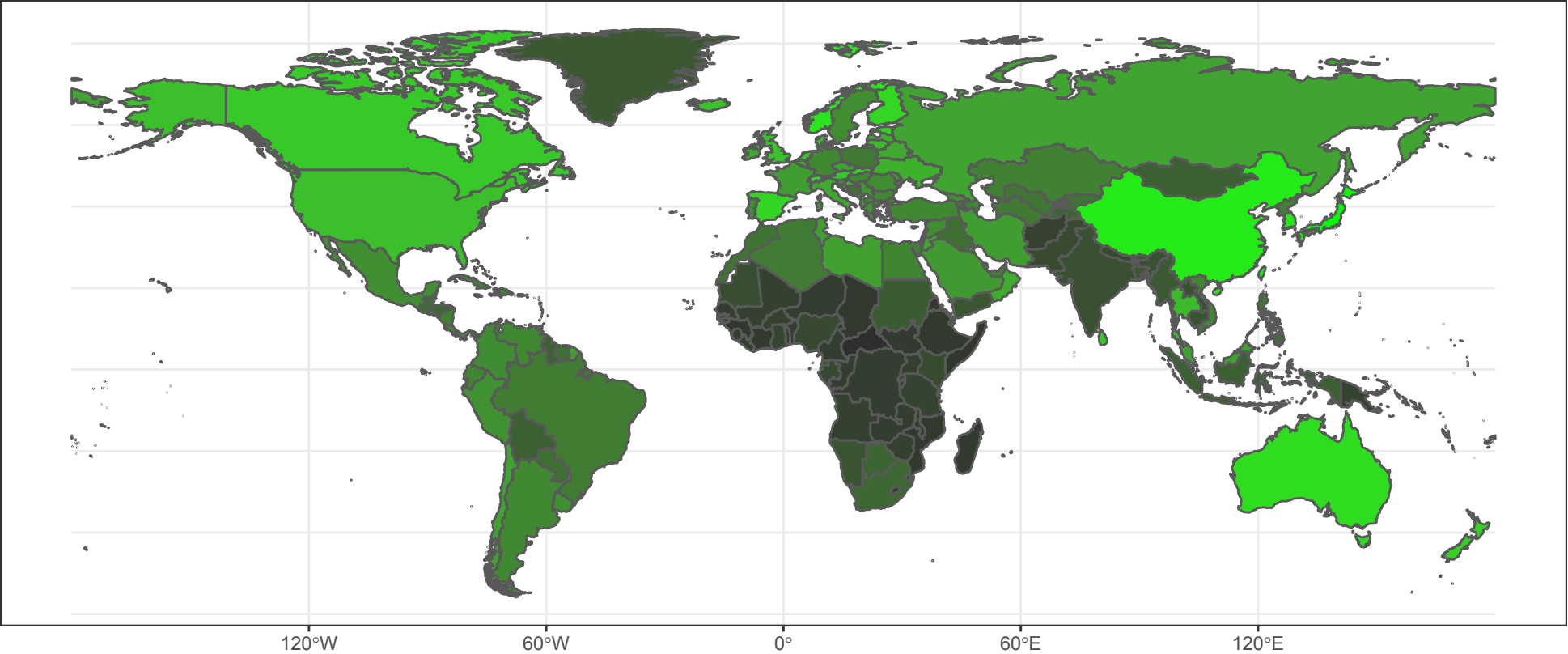

E

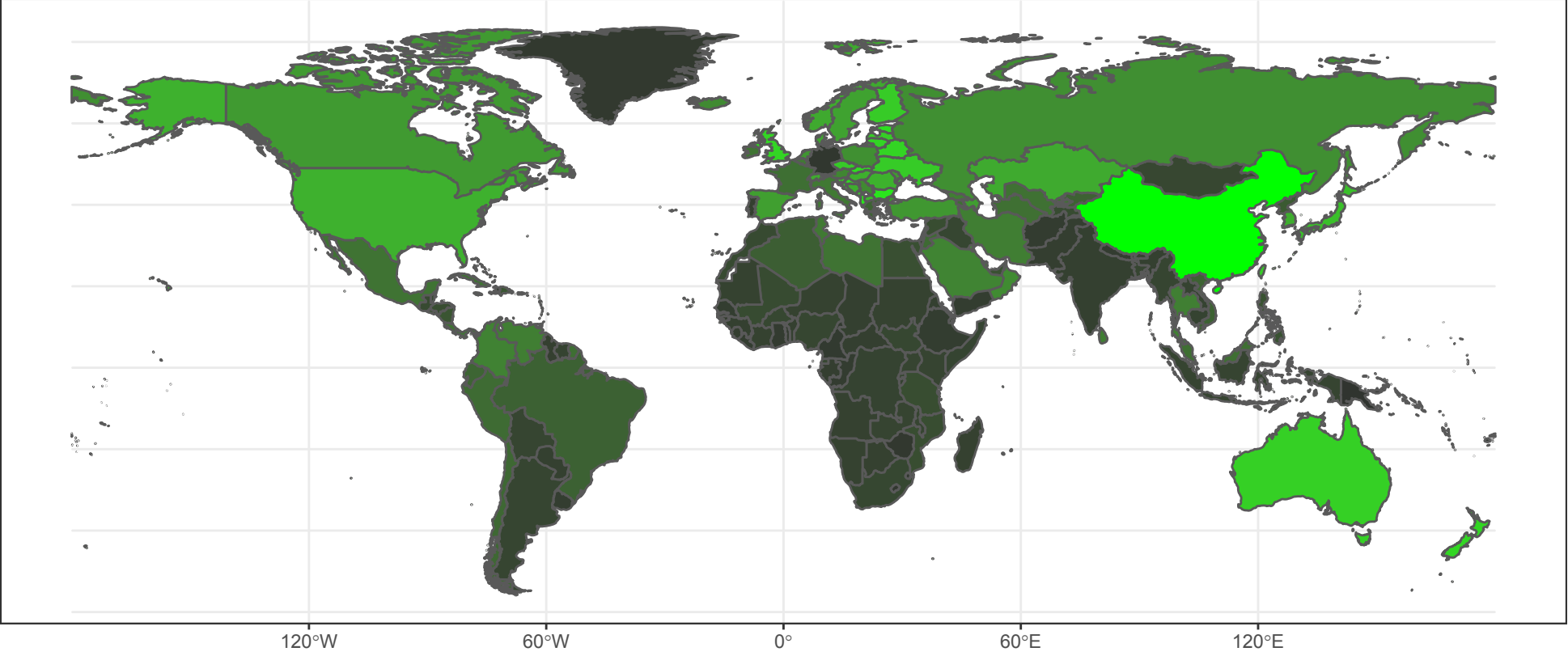

Supplement: Supplementary file 5 — Additional file 5: Figure S1. Age-standardized map of QCI scores at the national level, for AML (A), ALL (B), CML (C), CLL (D), and other leukemia (E), 2017. The QCI scores are illustrated on a scale of 0–100, so that higher scores represent better quality of care. Countries and territories are pictured by their QCI scores on a color-based scale where grey represents the lowest scores and green represents the highest scores. Abbreviations: ALL = acute lymphocytic leukemia. AML = acute myeloid leukemia. CLL = chronic lymphocytic leukemia. CML = chronic myeloid leukemia. QCI = Quality of Care Index. [file 40164_2021_198_MOESM5_ESM.pdf]

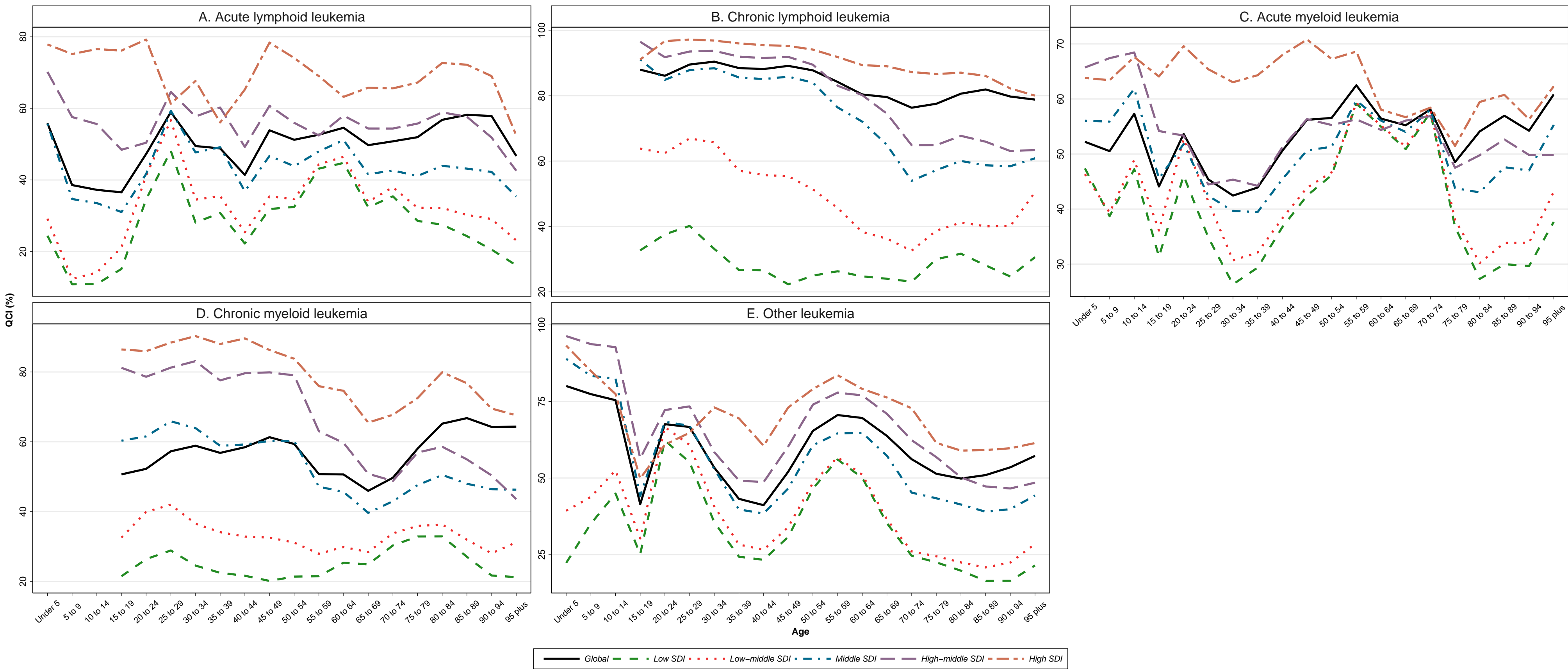

Supplement: Supplementary file 6 — Additional file 6: Figure S2. Range of QCI scores in different ages for AML (A), ALL (B), CML (C), CLL (D), and other leukemia (E), globally and by SDI quintile, 2017. The QCI scores are illustrated on a scale of 0–100, with 0 being the worst scores and 100 being the best scores. This figure demonstrates estimates for both sexes combined. Each line shows the association between QCI score and age in different areas including SDI quintile regions and global region. Abbreviations: ALL = acute lymphocytic leukemia. AML = acute myeloid leukemia. CLL = chronic lymphocytic leukemia. CML = chronic myeloid leukemia. QCI = Quality of Care Index. SDI = Socio-demographic Index. [file 40164_2021_198_MOESM6_ESM.pdf]

A

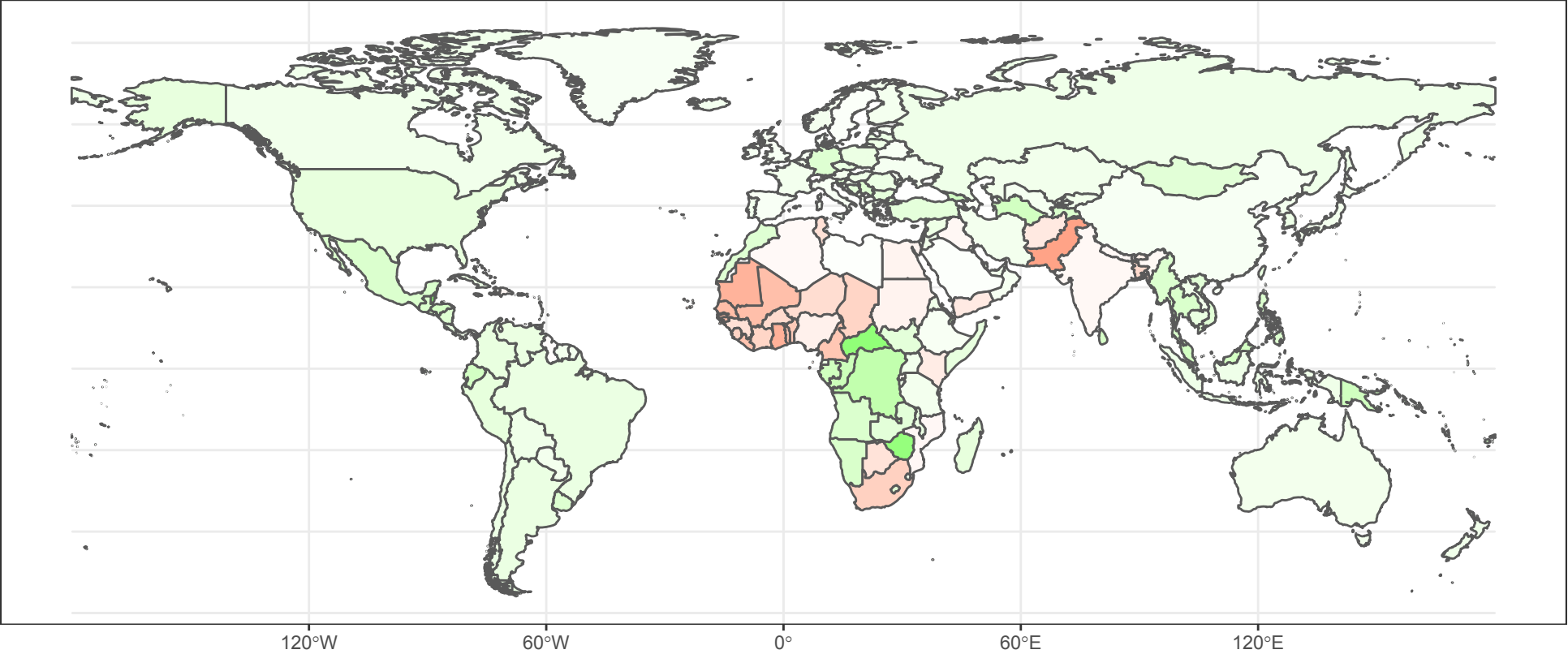

B

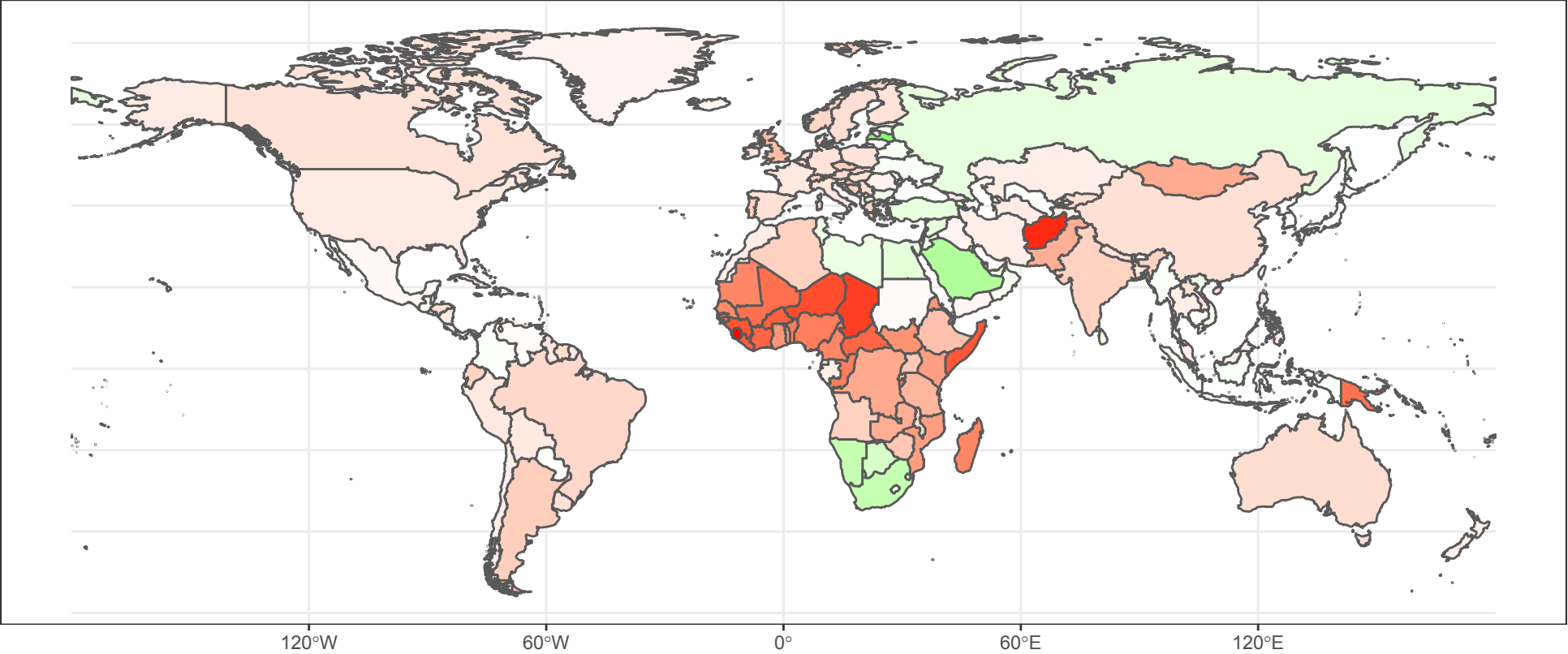

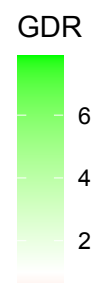

D

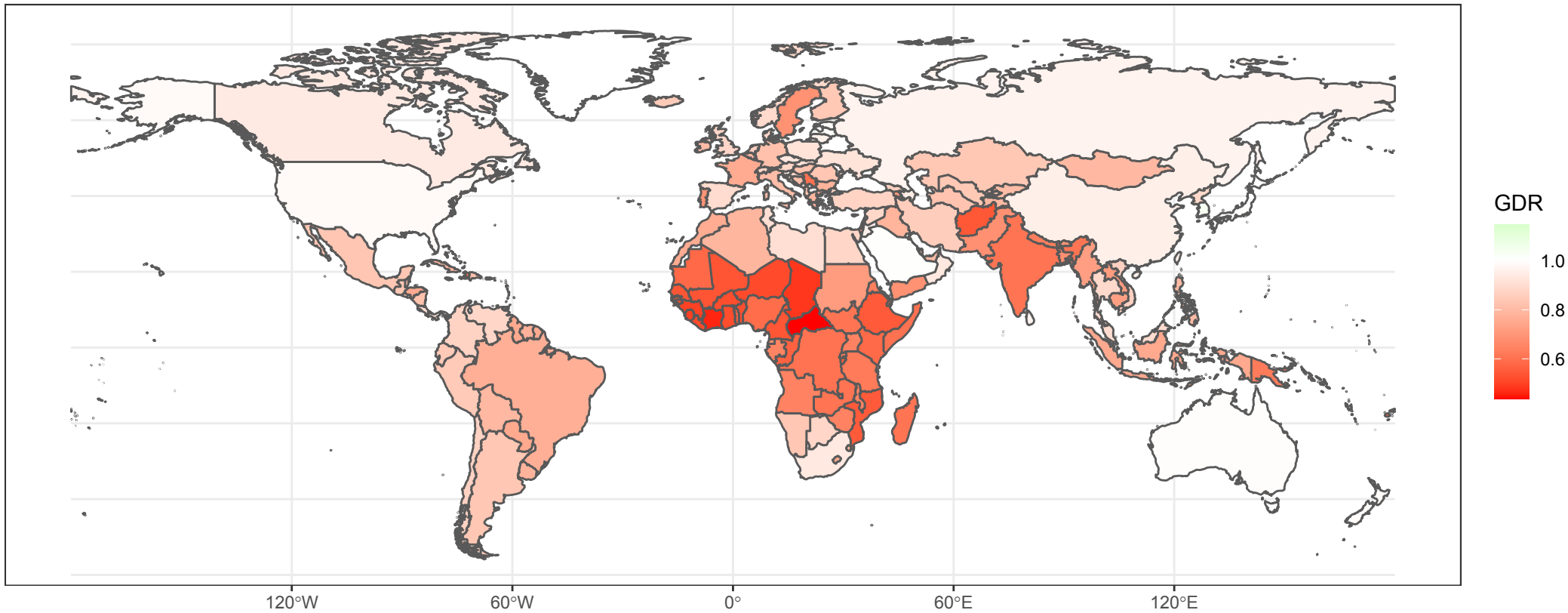

E

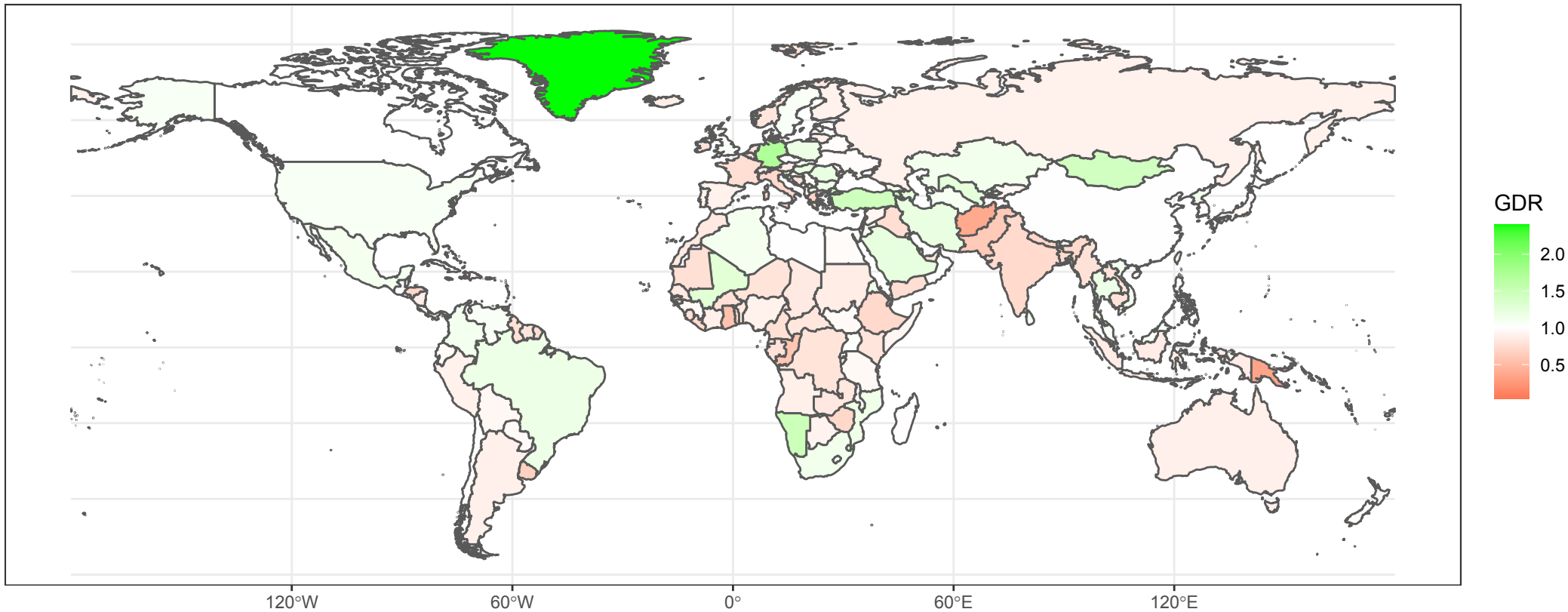

Supplement: Supplementary file 7 — Additional file 7: Figure S3. Age-standardized global map of gender disparity ratio, AML (A), ALL (B), CML (C), CLL (D), and other leukemia (E), 2017. The GDR is QCI score for females divided by QCI score for males, so that higher scores represent better care in females and lower scores represent better care in men. This figure illustrates the GDR in different countries and territories. Countries and territories are pictured by their GDR on a color-based scale where white represents the absence of disparity. Abbreviations: ALL = acute lymphocytic leukemia. AML = acute myeloid leukemia. CLL = chronic lymphocytic leukemia. CML = chronic myeloid leukemia. GDR = gender disparity ratio. QCI = Quality of Care Index. [file 40164_2021_198_MOESM7_ESM.pdf]

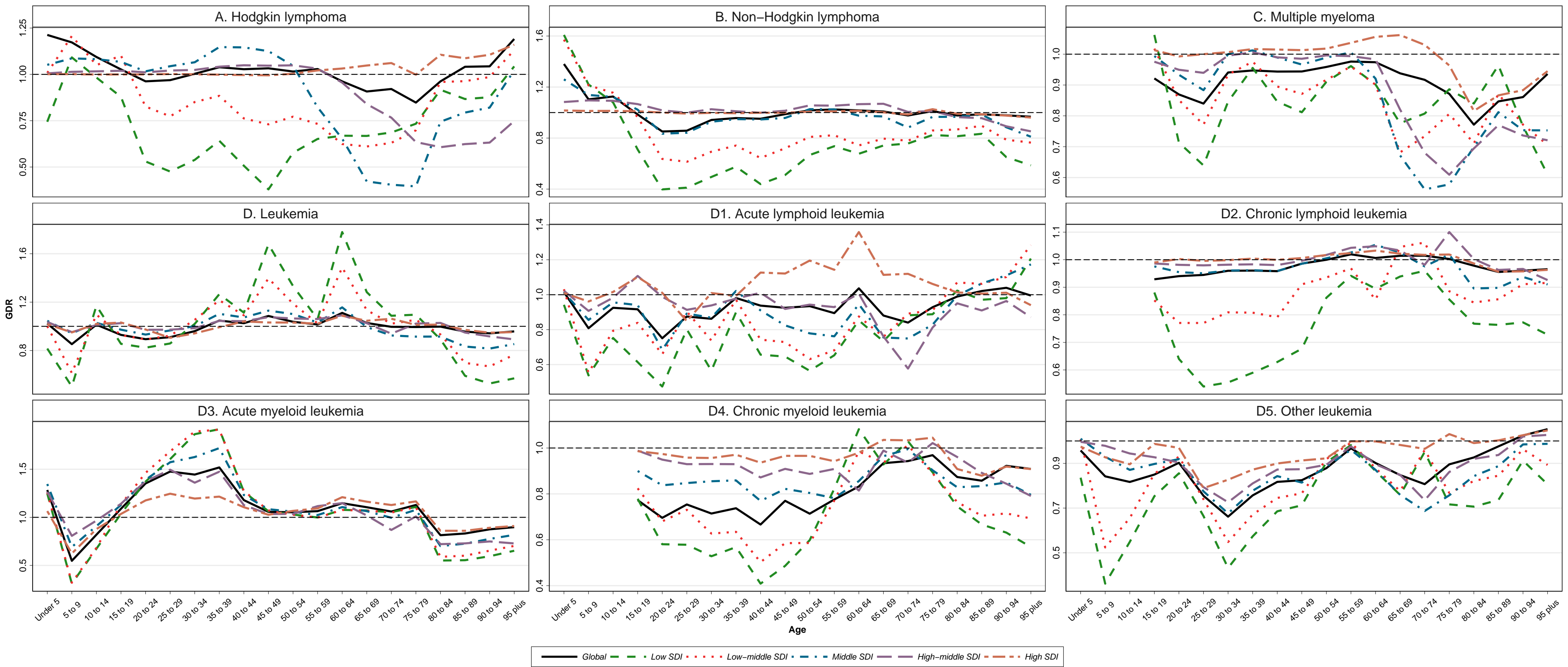

Supplement: Supplementary file 8 — Additional file 8: Figure S4. The association between gender disparity ratio and age for all hematologic malignancies globally and by SDI quintile regions in 2017. This figure demonstrates estimates for both sexes combined. Each line shows the association between GDR and age in different areas including SDI quintile regions and global region. Range of GDR is represented in nine figures for different subtypes of hematologic malignancies including HL (A), NHL (B), MM (C), leukemia (D), ALL (D1), CLL (D2), AML (D3), CML (D4), and other leukemia (D5). Abbreviations: ALL = acute lymphocytic leukemia. AML = acute myeloid leukemia. CLL = chronic lymphocytic leukemia. CML = chronic myeloid leukemia. GDR = gender disparity ratio. HL = Hodgkin lymphoma. MM = multiple myeloma. NHL = non-Hodgkin lymphoma. SDI = Socio-demographic Index. [file 40164_2021_198_MOESM8_ESM.pdf]
